# Association of Androgen-Receptor Gene Mutations with the Copy Number of Androgen-Receptor Silk Protein A Complex and Glutathione-S-Transferases T1 and M1 in Prostate Cancer Patients

**DOI:** 10.1155/2023/5956951

**Published:** 2023-02-14

**Authors:** Yan Zhang, Xiangdi Meng, Zhaosen Ma, Zhou Sun, Zhixin Wang

**Affiliations:** ^1^Department of Endocrinology, China-Japan Union Hospital of Jilin University, Changchun, Jilin 130022, China; ^2^Department of Urology, China-Japan Union Hospital of Jilin University, Changchun, Jilin 130022, China

## Abstract

**Objective:**

The purpose of our work was to explore the association of mutations in the androgen receptor gene and copy numbers of the androgen-receptor silk protein A complex with glutathione-S-transferases T1 and M1 in prostate cancer patients.

**Materials and Methods:**

Eighty-five patients with PC and 85 healthy controls were included in the study. Fasting peripheral venous blood was collected, whole blood genomic DNA was extracted, and AR gene-receptor genotype was detected by a high-resolution melting curve analysis detection technology. Expression levels of androgen receptor (AR) and filamin protein A (FlnA) were detected by Western blotting. RT-PCR was used to detect the copy number of T1 and M1 glutathione-S-transferases.

**Results:**

The wild-type androgen receptor gene rs5918762 is of TT type. The frequencies of CC and TC genes in the prostate cancer group were significantly higher than those in the normal control group (*P* < 0.05). Compared with TT-type PC patients, PC patients with TC-type and CC-type had higher expression levels of sex hormone receptor silk protein A complex and higher copy numbers of GSTT1 and GSTM1 (*P* < 0.05). Androgen-receptor gene mutation (T ⟶ C) was significantly positively correlated with the expression level of androgen-receptor silk protein A complex and the copy number of GSTT1 and GSTM1.

**Conclusion:**

Androgen-receptor gene polymorphisms were significantly associated with expression levels of androgen receptor complex *A* and silk proteins, and copy numbers of T1 and M1 glutathione-S-transferases. A combination of four factors can be used to identify prostate cancer susceptibility and disease progression.

## 1. Introduction

Androgen deprivation therapy (ADT) is the standard treatment for prostate cancer (PC) patients and is effective inhibiting the growth and progression of PC [1]. Tumor microenvironment consists of many stromal cells and inflammatory cells. Immunohistochemical (IHC) analysis shows that androgen/androgen receptor (AR) expression in a nonmalignant matrix is very high compared with the PC matrix, and a significant level of AR is detected in fibroblasts of human PC specimens [2]. Androgen-triggered androgen receptor/filaggrin A complex (AR/FlnA complex) drives the migration and invasion of fibroblasts [3]. Therefore, it is important to examine the relationship between genes encoding proteins and risk factor of individual susceptibility to cancer. Low-dose, long-term exposure to certain chemicals increases the risk of prostate cancer in general population [4].

Glutathione-S-transferases (GSTs) are a superfamily of phase II drug-metabolizing enzymes that convert reduced glutathione into a variety of electrophiles, including those found in smog, air pollution, and industrial chemicals. Catalyze conjugation expels toxins and protects cells from foreign substances [4]. Several human GST enzymes, such as Mu and Theta, are expressed in the human prostate tissue [3]. Therefore, mutations in the GST gene may be associated with PC.

Among the GST family genes, copy number changes have been found for GSTM1 and glutathione-S-transferase T1 (GSTT1). GSTM1 and GSTT1 polymorphisms are homozygous gene deletions leading to loss of GST enzymes [5]. Prostate is a tissue that responds to androgen. When the level of androgen increases, it is prone to prostate cancer. Individuals with one or two extra copies of GSTT1 gene may be particularly sensitive to prostate cancer, which is biologically reasonable. The aim of this study was to investigate whether mutations in GSTM1, T1, and the androgen-receptor gene and the androgen-receptor filamin protein A complex are associated with prostate cancer patients.

## 2. Materials and Methods

### 2.1. General Information

From April 2017 to April 2022, 85 patients with prostate cancer were enrolled in the Urology Department of China-Japan Friendship Hospital of Jilin University and are included in the prostate cancer group. The normal controls were 85 healthy males who received physical examination in the physical examination center of our hospital. Prostate cancer group inclusion criteria are as follows: age ≥18 years, hospital residents, and holistic care. Those who provided written informed consent had no genetic link to the prostate patients.

Patients with other types of cancer, severe cardiopulmonary disease, or comorbidities for which valid and reliable data were available and were excluded from this study. After admission, trained interviewers conducted structured face-to-face interviews with all participants. Detailed medical information on demographics, social and lifestyle characteristics, family history of cancer, and PC were assessed. All participants gave informed consent.

### 2.2. Methods

#### 2.2.1. Genomic DNA Extraction

In the morning, 5 mL of peripheral venous blood was taken from the two groups of subjects on an empty stomach, placed in EDTA anticoagulation centrifuge tube, and stored in the refrigerator at −40°C for later use. The whole blood genomic DNA was extracted by Qiagen DNA purification kit, and eluted to 200 *μ*L. The integrity of DNA was identified by 1% agarose gel electrophoresis, the concentration was measured by a UV-vis spectrophotometer, and the purity of DNA was evaluated by OD260/OD280. After detection, the whole blood genome DNA was complete, the concentration was in the range of 80∼100 ng/mL, and the OD260/OD280 was in the range of 1.7∼1.8, which met the requirements of subsequent experiments.

#### 2.2.2. Detection of Androgen Receptor Gene Mutation

The genotype of AR gene receptor was detected by a high-resolution melting curve analysis. Firstly, the SNP database of NCBI (https://www.ncbi.nlm.nih.gov/snp/) screened out the inclusion site rs5918762, with the minimum allele frequency of 3′UTR in Chinese population >5% and polymorphic linkage relationship of <0.6. Secondly, primers covering the sequence of rs5918762 (forward: 5′-GTGAGGACGGGCTGTAGAAG-3′ and reverse: 5′-GTGAGGACGGGCTGTGAAG-3′) were designed by NCBI primer-BLAST, and PCR amplification was performed (predenaturation at 94°C for 5 minutes, 95°C for 35 seconds, and 56°C for 30 seconds). At last, PCR amplification products were mixed with fluorescent dyes, and then, PCR amplification was carried out (95°C for 5 minutes, 25°C for 30 seconds, and then stored at 4°C). After centrifugation at 4000 rpm for half an hour, the genotype of rs5918762 was determined by the high-resolution melting curve analysis and detection system. The PCR products from the first step were tested by the Sanger method.

#### 2.2.3. Western Blotting Analysis of Androgen Receptor/Silk Protein A Complex

Whole blood total protein was extracted with RIPA buffer and stored at 80°C. Protein volumes loaded onto polyacrylamide gel electrophoresis were normalized to *β*-actin and then separated on 8% sodium dodecyl sulfate polyacrylamide gel electrophoresis at 90 V for 90 min. Proteins were transferred to PVDF membranes using primary antibodies (anti-AR; anti-FlnA;anti-*β*-actin) and secondary IgG. Then, the blot was visualized using Image *J*.

#### 2.2.4. RT-PCR Was Used to Detect the Copy Number of Glutathione-S-Transferases T1 and M1

The primers (FAM probes) and probe sequences of GSTT1 and GSTM1 used in the RT-PCR detection experiment are shown in Table 1. Endogenous ribonuclease P (RNase P, VIC probe labeling) was set as the internal reference control.

The reaction system of RT-PCR consisted of 12.5 *μ*L of 2 × TaqMan PCR mixture, 1 *μ*L of FAM probe, 0.5 *μ*L of positive and negative sequences of primers, 0.2 *μ*l of DNA, and 25 *μ*L of ddH2O. The reaction conditions were as follows: 48°C for 2 minutes, 92°C for 10 minutes, 92°C for 30 seconds, and 58°C for 40 seconds, with 40 cycles.

The calculation method was as follows: CT target gene = 2 × 2^−ΔΔCt^, where ΔΔCt = ΔCtGST sample −ΔCtRNase *P* and ΔCt was the average CT value of three repeated measurements of the sample. The CT value was the relative copy number of GSTT1 and GSTM1 genes and RNase *P* of internal reference control.

### 2.3. Statistical Analysis

SPSS 24.0 statistical software was used in this study. Results of data analysis are presented as mean ± standard error. Data analysis between two groups was performed by the *t*-test. Subsequent analyzes were performed using the LSD test. The Pearson correlation test was performed to analyze the correlation between the two parameters. *P* ≤ 0.05 is considered as statistically significant.

## 3. Results

### 3.1. General Condition of the Subjects

Baseline data for subjects in the study are shown in Table 2.

### 3.2. Mutation of Androgen-Receptor Gene in the Subjects of Two Groups

**Figure 1:**
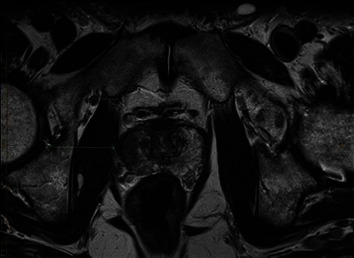
MRI image of prostate in patients with prostate cancer.

**Figure 2:**
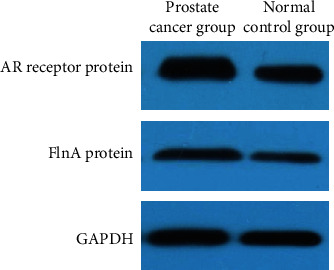
Expression of androgen receptor silk protein A complex. *Note*. 1–3 are samples of the normal control group; 4–6 are samples of the prostate cancer group.

There were gene locus changes in the prostate cancer group and normal control group. Hardy–Weinberg equilibrium test results of rs5918762 locus changes are shown in Table 3. There were significant differences in cell viability, SOD activity, and miR-146a expression between the two groups. However, the LDH activity, MDA, GRP78 levels, CHOP expression, and cell apoptosis rate in the H/Ri + Dex group were lower than those in the HR group. The results are shown in Table 3 and Figure 1. Therefore, dexmedetomidine could upregulate miR-146a and reduce oxidative stress and ERS in H/Ri-transformed rat cardiomyocytes, thereby increasing cell viability and reducing the rate of apoptosis (see Figure 2).

### 3.3. Expression of Androgen-Receptor Silk Protein A Complex in the Subjects of Two Groups

We also measured the expression level of androgen-receptor silk protein complex A in both the groups. The results showed that the relative expression of AR/FlnA complex in the PC group was significantly higher than that in the normal control group, see Table 4.

### 3.4. Genotype Distribution of Glutathione-S-Transferases in the Subjects of Two Groups

Genotypes of glutathione-S-transferases GSTT1 and GSTM1 in the two groups are shown in Table 5. In the control group, the number of subjects with copies of GSTT1 gene 0, 1, 2, and > 2 was 37, 34, 11, and 3, respectively, and the number of subjects with copies of GSTM1 gene 0, 1, 2, and > 2 was 55, 16, 9, and 5, respectively. In the prostate cancer group, the number of subjects with GSTT1 gene copy numbers of 0, 1, 2, and > 2 was 33, 25, 16, and 11, respectively, and the number of subjects with GSTM1 gene copy numbers of 0, 1, 2, and > 2 was 46, 20, 10, and 9, respectively. There was a statistically significant difference in the prostate cancer group with more than two copies of the GSTT1 and GSTM1 genes compared to the normal control group. The results showed that men with two or more gene copy number changes had a significantly higher risk of developing prostate cancer.

### 3.5. Association between Androgen-Receptor Gene Mutation and Copy Numbers of Androgen Receptor Silk Protein A Complex and Glutathione-S-Transferases T1 and M1 in Patients with Prostate Cancer

Significant differences in the expression level of androgen-receptor silk protein complex and the copy number of glutathione-S-transferases T1 and M1 in patients with three genotypes of PC (*P*  < 0.05). The results are shown in Table 6.

### 3.6. Correlation Analysis

Pearson correlation test showed that the mutation of androgen receptor gene (T ⟶ C) was positively correlated with the expression level of androgen-receptor silk protein complex A and the copy numbers of GSTT1 and GSTM1. The results are shown in Tables 7 and 8.

## 4. Discussion

During the progress of cancer, malignant cells will accumulate somatic mutations, which will lead to gene abnormalities [6]. Genetic and environmental interactions promote PC development. Among genetic factors, the role of AR as a potential risk factor for PC has received special attention. Understanding the contribution of AR polymorphisms and their interactions with other relevant factors may improve screening and computational analyses.

Androgen action requires proper signal transmission through nuclear transcription factor AR. Originally located in the cytoplasm, it should be translocated to the nucleus to interact with DNA [7]. Filamin A, a protein encoded by FLNA gene, is a coactivator of many cytoplasmic factors (including AR). Androgen-triggered AR/FlnA complexes can drive the migration and invasion of CAFs and prevent the assembly of AR/FlnA complexes in androgen-treated CAFs, which can effectively inhibit these reactions [8].

In this study, we found that CAG repeat length can serve as a useful marker to identify clinically relevant prostate cancer risk groups [9]. CAG repeats may be associated with TMPRSS2: ERG fusion-positive prostate cancer, but may be protective against PC in China [10].

Recent studies have assessed the combined effects of GSTM1 and GSTT1 genotypes, but few studies have shown a significant association between the defects in these genes and prostate cancer risk [11–13]. Genes involved in oncogenic activation or detoxification have not yet been isolated, and more number of genes need to be assessed to fully understand this phenomenon. M1 and T1 glutathione-S-transferases are being investigated as risk candidates for smoking-related cancers. Several reports proposed adding PC to the list of cancers for which smoking is a risk factor and investigated possible associations between GSTM1 and GSTT1 deficient genotypes and susceptibility to PC in smokers [14, 15]. The use of molecular biomarkers such as GST in the management of CP patients may improve clinical outcomes. Therefore, we believe that understanding the potential role of GST copy number in CP risk can make a significant contribution to the field. Evidence from this study demonstrated that there is an association between AR genotype and the risk of PC, and that AR-CC genotype increases an individual's susceptibility to prostate cancer.

In this study, TC-type and CC-type prostate cancer patients had higher expression level of androgen-receptor filamin A complex and higher copy numbers of GSTT1 and GSTM1. This finding was confirmed by related studies. Men with a GSTM1 deletion genotype, a GSTM1 and GSTT1 double deletion genotype, or a GSTT1 deletion gene and the GSTP1-A131 G polymorphism have an increased risk of developing prostate cancer [16]. Double null genotype GSTM1/GSTT is associated with a higher risk of prostate cancer in Asians [17, 18].

There were some limitations in this study when interpreting the results. The number of prostate cancer researchers with less than 100 cases limited the ability to study the association with androgen-receptor gene mutation, and the influence of low penetrance gene (such as GST) polymorphism needed hundreds of patients to be recognized. Since the study population is Chinese, the GSTM1 and GSTT1 gene copy number results cannot be extrapolated to other races/ethnicities. Furthermore, these patients were relatively young (40–64 years) at diagnosis, so this finding may not apply to older men. Another potential limitation is the lack of studies on prostate cancer recurrence/progression, which requires further research. The reason why this study is conducted with a small sample size is that the current number of subjects can meet the needs of the study through sample size calculation. Also, mutations in the androgen receptor gene are uncommon in men with prostate cancer in the general population, so it would take a long time to get a large sample. Long-term use of medications may impact the risk of diseases [19, 20]. The interaction between taking drugs and genes needs to be considered. Vitamin D deficiency is associated with PC [21] and diabetes [22]. This study did not examine the interaction of diet (e.g., vitamin D intake) and genetics on PC. Physical activity might play a protective role against PC development [23, 24]. The role of interaction between physical activity and gene on PC should be explored. The number of subjects does not reach hundreds, which is also the limitation of this study. In future studies, we will continue to collect androgen-receptor gene mutations in prostate cancer patients, aiming to conduct multicenter studies to improve the representativeness of the results of this study. In addition, this study did not analyze GSTP1 gene, GSTM1, and GSTT1, which will be tested in subsequent studies to explore the clinical value of more gene mutation sites.

## 5. Conclusion

Taken together, the results indicated that androgen-receptor gene polymorphisms were significantly associated with the expression levels of androgen receptor silk protein A complex and the copy numbers of T1 and M1 glutathione-S-transferases. As prognostic biomarkers, these four factors can be used together to determine PC susceptibility and disease progression.

## Figures and Tables

**Table 1:** Primer and probe sequence.

Gene name	Type	Primer sequence
GSTM1	Primer	Positive: 5′-CACCTGCATTCGTTCATGTGAC-3′ and inverse: 5′-CAGAGAGGG-AGGGGCACTCA-3′
	Hybridization probe	6FAM-TTCAGTCCTGCCATGAGCAGGCACA-TAMRA

GSTT1	Primer	Positive: 5′-AAGTCCCAGAGCACTCACCTCC-3′ and inverse: 5′-CAGTGCATC-ATTCTCTCATCTGGC-3′
	Hybridization probe	6FAM-CACCATCCCCACCCTGTCTTCCCA-TAMRA

**Table 2:** Baseline data for subjects in the study (*n*, %).

Project		Prostate cancer group (*n* = 85)	Normal control group (*n* = 85)	*χ* ^2^ value	*P* value
BMI (kg/m^2^)	≤30	54 (63.53)	58 (68.24)	0.419	0.518
	>30	31 (36.47)	27 (31.76)		

Age	≤65	38 (77.71)	31 (36.47)	1.195	0.274
	>65	47 (55.29)	54 (63.53)		

History of smoking	Yes	62 (72.94)	51 (60.00)	3.194	0.074
	No	23 (27.06)	34 (40.00)		

**Table 3:** Hardy–Weinberg equilibrium test (*n*, %).

Genotype	Prostate cancer group		Normal control group	
	Sanger test	HRMCAD	Sanger test	HRMCAD
TT type	56 (65.88)	53 (62.35)	77 (90.59)	75 (88.24)
CT type	23 (27.06)	28 (32.94)	8 (9.41)	10 (11.76)
CC type	6 (7.06)	4 (4.71)	0 (0.00)	0 (0.00)

*χ * ^2^ value	0.273		1.150	
*P* value	0.601		0.446	

HRMCAD: high-resolution melting curve analysis and detection.

**Table 4:** Expression of AR/FlnA complex between the subjects of two groups (/*β*-actin antibody).

Groups	AR receptor protein expression level	FlnA protein expression level
Prostate cancer group (*n* = 85)	1.47 ± 0.14	3.86 ± 0.52
Normal control group (*n* = 85)	0.63 ± 0.11	3.24 ± 0.47
*t* value	43.497	8.155
*P* value	<0.001	<0.001

**Table 5:** Genotype distribution of GST in the subjects of two groups.

Gene name	Copy number	*N* (%)		*P* value	OR (95% CI)
		Normal control group	Prostate cancer group		
GSTT1	0	37 (43.53)	33 (38.82)	0.048^*∗*^	1.56 (1.00–2.38)
	One	34 (40.00)	25 (29.41)	0.063^#^	0.54 (0.35–1.01)
	2	11 (12.94)	16 (18.82)		1 (reference group)
	>2	3 (0.35)	11 (12.94)	0.023^*∗*^	0.43 (0.18–1.17)

GSTM1	0	55 (64.71)	46 (54.12)	0.032^*∗*^	0.86 (0.55–1.24)
	One	16 (18.82)	20 (23.53)	0.41^#^	0.79 (0.42–1.56)
	2	9 (10.59)	10 (11.76)		1 (reference group)
	>2	5 (0.59)	9 (10.59)	0.034^*∗*^	0.83 (0.32–2.37)

*Note.* ^*∗*^compared with the remaining three genotype combinations; ^#^compared with the genotype combination with copy number 2.

**Table 6:** Comparison of AR/FlnA expression and copy number of GST gene in different genotypes of prostate cancer patients at rs5918762 locus.

Genotype	AR/FlnA (/*β*-actin)		GSTT1 copy number		GSTM1 copy number	
	<4	≥4	<2	≥2	<2	≥2
TT type (*n* = 56)	32 (57.14)	24 (42.86)	54 (96.43)	2 (3.57)	55 (98.21)	1 (0.18)
CT type (*n* = 23)	8 (34.78)	15 (65.22)	3 (13.04)	20 (86.96)	11 (47.83)	12 (52.17)
CC-type (*n* = 6)	2 (33.33)	4 (66.67)	1 (16.67)	5 (83.33)	0 (0.00)	6 (100.00)

*χ* ^2^ value	7.556		9.035		9.971	
*P* value	0.018		0.008		0.004	

**Table 7:** Gene matrix of the prostate cancer group and normal control group.

No	Androgen-receptor gene mutation	Number of GSTT1 copies	Number of copies of GSTM1	AR-protein expression level	FlnA-protein expression level
1	CT	3	4	1.99	3.79
2	CT	3	3	1.85	3.85
3	CT	3	3	1.55	3.96
4	CT	3	3	1.76	4.02
5	CT	3	1	1.51	3.88
6	CT	4	3	1.69	3.92
7	CT	3	3	2.01	4.10
8	CT	4	2	1.67	3.88
9	CT	2	2	1.72	3.96
10	CT	2	2	1.52	4.03
11	CT	2	2	1.63	3.86
12	CT	2	2	1.75	3.95
13	CT	2	2	1.68	3.96
14	CT	3	2	1.62	3.90
15	CT	3	2	1.75	4.01
16	CT	2	2	1.83	3.92
17	CT	2	1	1.62	3.99
18	CT	2	1	1.69	3.76
19	CT	2	1	1.53	3.88
20	CT	2	1	1.49	3.75
21	CT	2	1	1.92	3.70
22	CT	2	1	1.56	3.69
23	CT	1	1	1.68	3.69
24	CT	1	1	1.78	3.75
25	CT	2	1	1.75	3.65
26	CT	1	2	1.83	2.10
27	CT	1	1	1.82	2.05
28	CT	1	1	1.61	1.96
29	CC	2	1	1.66	1.85
30	CC	2	4	1.88	1.93
31	CC	3	3	1.69	1.72
32	CC	2	2	1.55	1.66

**Table 8:** Correlation analysis.

Correlation index	Androgen-receptor gene mutation	
	*r*	*P*
The expression level of AR/FlnA complex	0.532	<0.001
GSTT1 copy number	0.507	<0.001
GSTM1 copy number	0.546	<0.001

## Data Availability

The datasets generated and analyzed during the current study are available from the corresponding author on reasonable request.
